# Outcomes of bacillus Calmette–Guérin therapy without a maintenance schedule for high‐risk non‐muscle‐invasive bladder cancer in the second transurethral resection era

**DOI:** 10.1111/iju.14761

**Published:** 2021-12-11

**Authors:** Hiroshi Kikuchi, Takashige Abe, Ryuji Matsumoto, Takahiro Osawa, Satoru Maruyama, Sachiyo Murai, Nobuo Shinohara

**Affiliations:** ^1^ Department of Urology Hokkaido University Graduate School of Medicine Japan; ^2^ Department of Urology National Hospital Organization Hokkaido Cancer Center Sapporo, Hokkaido Japan

**Keywords:** bacillus Calmette–Guérin, induction therapy, non‐muscle‐invasive bladder cancer, second transurethral resection

## Abstract

**Objectives:**

We examined the outcomes of eight weekly bacillus Calmette–Guérin induction therapy after second transurethral resection, and investigated risk factors for intravesical recurrence or disease progression in high‐risk non‐muscle‐invasive bladder cancer patients.

**Methods:**

This retrospective study included 146 high‐risk non‐muscle‐invasive bladder cancer patients who received eight weekly bacillus Calmette–Guérin instillations without a maintenance schedule between 2000 and 2019. Intravesical recurrence‐free and progression‐free survival rates were evaluated using the Kaplan–Meier method. The Cox proportional hazards model was used to identify risk factors.

**Results:**

Pathological T staging in the first transurethral resection was pTa in 56 patients (38.4%), pT1 in 75 (51.4%) and primary carcinoma *in situ* in 15 (10.2%). A total of 109 (83.2%) with pTa–1 disease underwent second transurethral resection before bacillus Calmette–Guérin induction therapy, and residual disease was detected in 54 (49.5%). The completion rate of eight instillations was 82.2%. The 2‐ and 5‐year intravesical recurrence‐free survival rates were 80.7% and 75.2%, whereas the 2‐ and 5‐year progression‐free survival rates were 85.7% and 82.0%. Recurrent tumors (hazard ratio 6.5830, *P* = 0.0007) and residual tumors at the second transurethral resection (hazard ratio 4.0337, *P* = 0.0021) were risk factors for intravesical recurrence. Multiple tumors (hazard ratio 5.8056, *P* = 0.0302), pT1 disease (hazard ratio 3.7351, *P* = 0.0447) and residual tumors at second transurethral resection (hazard ratio 3.2552, *P* = 0.0448) were associated with disease progression.

**Conclusions:**

Accurate disease staging and disease elimination by second transurethral resection followed by eight weekly bacillus Calmette–Guérin instillations achieved good disease control. Our protocol (without a maintenance schedule) after thorough surgical resection has potential as a treatment option in the current bacillus Calmette–Guérin shortage.

**Abbreviations & Acronyms**
AE: adverse event
BCG: bacillus Calmette–Guérin
CIS: carcinoma *in situ*
CTCAE: Common Terminology Criteria for Adverse Events
EAU: European Association of Urology
HR: hazard ratio
NMIBC: non‐muscle‐invasive bladder cancer
PFS: progression‐free survival
RFS: recurrence‐free survival
TUR: transurethral resection

## Introduction

NMIBC accounts for approximately 75% of initially diagnosed bladder cancers.^1^ NMIBC can be risk stratified into low‐, intermediate‐ and high‐risk groups based on the probability of tumor recurrence or progression, and high‐risk NMIBC is defined by the EAU guidelines as a T1 tumor, CIS, high‐grade (G3) tumor or TaG1/G2 tumor with multiple, recurrent and large (>3 cm) tumors.^2^ The standard treatment for high‐risk NMIBC patients is TUR followed by intravesical instillations with BCG.^3^, ^4^ In terms of BCG therapy, since the Southwest Oncology Group reported significant benefits for RFS with standard induction followed by a 3‐year maintenance BCG schedule over BCG induction alone, a maintenance schedule has now become widespread worldwide.^5^ However, just 16% of patients completed their planned schedule due to significant toxicity,^5^ and to mitigate BCG‐related AEs while maintaining treatment benefits, several clinical trials have been undertaken with a reduced number of BCG maintenance instillations.^6^, ^7^ The majority of studies carried out to date have mainly focused on the BCG maintenance schedule or BCG dose, and not on the quality of TUR before BCG induction therapy.^8^, ^9^

To identify residual disease and confirm the non‐muscle‐invasive disease status, a second TUR is recommended for high‐risk NMIBC patients by the EAU guidelines.^2^ Our group also actively carries out second TUR on high‐risk pTa/T1 bladder cancer patients before BCG induction therapy. We have not yet used maintenance BCG therapy. In the present study, we retrospectively evaluated the treatment outcomes of BCG therapy in a cohort in which a large proportion underwent second TUR, and investigated risk factors for intravesical recurrence and disease progression.

## Methods

### Study population

Between January 2000 and September 2019, 432 patients underwent TUR for bladder cancer at Hokkaido University Hospital, Sapporo, Japan. A total of 223 patients (52%) were diagnosed with high‐risk NMIBC, defined by the EAU guidelines as a T1 tumor, CIS, a high‐grade (G3) tumor or TaG1/G2 tumor with multiple, recurrent and large (>3 cm) tumors.^2^ Of these high‐risk NMIBC patients, 153 (69%) received intravesical BCG (Tokyo‐172 or Connaught strain) therapy and this cohort was our interest. Excluding four patients receiving second‐line BCG and three patients with <6 months follow up, 146 were included in the present study. Institutional review board approval was obtained from Hokkaido University Hospital (019‐0092). Patient characteristics, including age, sex, tumor status (primary or recurrent), tumor number, tumor size, pathological outcomes, urine cytology and AEs with BCG, were retrospectively collected from our prospectively maintained database and medical charts. AEs were graded according to the CTCAE Version 5.0.^10^

### Treatment and follow‐up protocol

A second TUR was actively carried out within 3–12 weeks of initial TUR, with the aims of eliminating disease and obtaining a correct diagnosis to rule out muscle‐invasive disease. All visible tumors and resection scars or edematous areas from initial TUR sites were resected at the second TUR. The margin for a second TUR included one loop width lateral to the original TUR sites, and deep muscle specimens were also collected from the previous tumor areas. Patients with high‐grade Ta/T1 tumors were indicated for a second TUR; however, this was sometimes omitted at the discretion of the attending physician; for example, based on the status of the patient (old age or comorbidities).

The instillation of BCG was carried out approximately 1 month after TUR. Lyophilized BCG (80 mg of Tokyo‐172 or 81 mg of Connaught) was suspended in 40 mL of normal saline. After catheterizing the bladder, the suspension was instilled, the catheter was withdrawn, and patients were requested to retain the liquid for 1–2 h. A half dose of BCG was also utilized in some patients due to old age or comorbidities (*n* = 6). After BCG induction, cystoscopy and urine cytology were carried out every 3 months during the first 2 years and every 6 months thereafter. Protocol biopsy was generally considered 6 months after the initiation of BCG, and punch biopsies were carried out on the right and left lateral sides, the dome, anterior, and trigone, as well as on any suspicious areas under lumbar or general anesthesia. Prostatic urethra biopsy was added based on the decisions of surgeons.

### Statistical analysis

Intravesical recurrence and PFS rates were estimated using the Kaplan–Meier method. The time to recurrence or progression was defined as the time from the date of the first TUR to that of the first intravesical recurrence or disease progression. Intravesical recurrence was defined as any type of recurrence in the bladder. The follow up of patients without bladder tumor recurrence was censored to the date of their last visit. Disease progression was defined as an increase in the T stage on TUR, development of metastatic disease, cystectomy or bladder cancer‐related death. In the case of death not related to bladder cancer, the follow up was censored to the date of death. A logistic regression analysis was used to identify factors associated with residual tumors at the second TUR. Survival rates were compared by the log‐rank test, and the Cox proportional hazards regression model was used to identify factors associated with intravesical recurrence or disease progression. Factors examined included age, sex, tumor status (primary or recurrent), tumor number, tumor size, pathological outcomes (T stage and concurrent CIS) and residual tumors at the second TUR. jmp version 14 (SAS Institute, Cary, NC, USA) was used for all calculations, and *P* < 0.05 was considered to be significant.

## Results

**Fig. 1:**
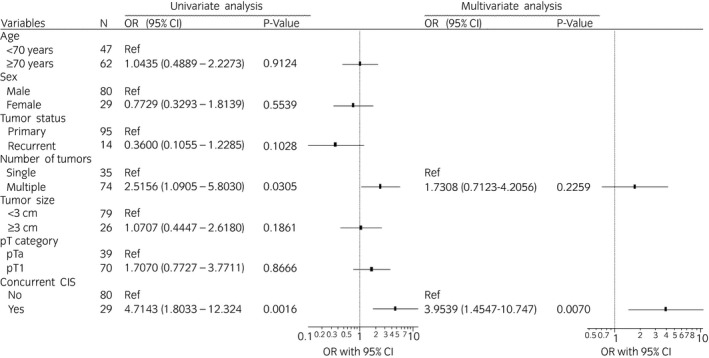
Univariate and multivariate logistic regression analyses to predict residual tumors at second TUR.

The clinical and pathological features of the 146 patients examined are shown in Table 1. Median age was 72 years (interquartile range 65–77 years), and 74.7% of patients were men. Pathological T staging at first TUR was pTa in 56 (38.4%) patients, pT1 in 75 (51.4%) and primary CIS in 15 (10.2%). Concurrent CIS was detected in 44 (33.6%) patients with pTa–1 disease. Overall, 109 (83.2%, 109/131) patients with pTa–1 disease underwent second TUR before BCG induction therapy. Among patients with pT1 disease, 93.3% (70/75) underwent second TUR. Residual tumors were detected in 16 (41.0%) patients with Ta and in 39 (54.3%) with T1 disease (Table 2). Univariate and multivariate analyses identified concurrent CIS as an independent risk factor for residual tumors at second TUR (*P* = 0.0070, Fig. 1).

**Table 1:** Baseline characteristics for patients and disease

Variables	
Median age at BCG instillation, years (interquartile range)	72 (65–77)
Sex, *n* (%)	
Male	109 (74.7)
Female	36 (25.3)
Primary or recurrent, *n* (%)	
Primary	115 (78.8)
Recurrent	31 (21.2)
Tumor multiplicity, *n* (%)	
Single	44 (30.1)
Multiple	102 (69.9)
Tumor size, *n* (%)	
<3 cm	115 (78.8)
≥3 cm	27 (18.5)
Unknown	4 (2.7)
pT stage at first TUR, *n* (%)	
pTa	56 (38.4)
pT1	75 (51.4)
Primary CIS	15 (10.2)
Concurrent CIS†, *n* (%)	44 (33.6)
Second TUR†, *n* (%)	
Yes	109 (83.2)
In Ta	39 (69.6)
In T1	70 (93.3)
No	22 (16.8)
Residual tumor at second TUR, *n* (%)	
Yes	54 (49.5)
In pTa	16 (41.0)
In pT1	38 (54.3)
No. BCG instillations	
8	120 (82.2)
7	6 (4.1)
6	12 (8.2)
≤5	8 (5.5)
BCG strain used, *n* (%)	
Tokyo‐172‡	128 (87.7)
Connaught	18 (12.3)
Median follow up, months (range)	61 (9–207)

† Second TUR and associated CIS rates were counted in Ta/T1 cases.

‡ Six patients received a half dose of Immunobladder. Total *n* = 146.

**Table 2:** Pathological results of second TUR

	Overall (*n* = 109)	T stage at first TUR	
		Ta (*n* = 39)	T1 (*n* = 70)
T stage at second TUR, *n* (%)			
No residual	55 (50.5)	23 (59.0)	32 (45.7)
CIS	16 (14.7)	5 (12.8)	11 (15.7)
pTa	27 (24.8)	11 (28.2)	16 (22.9)
pT1	11 (10.1)	0 (0.0)	11 (15.7)

Total *n* = 109.

The completion of six or eight weekly instillations of BCG were 94.5 or 82.2%, respectively. Tokyo‐172 was the dominant BCG strain (87.7%, 128/146). Regarding AEs with BCG, six (4.1%) patients required hospitalization for toxicity (CTCAE grade 3), and 75 (51.8%) received oral medication (CTCAE grade 2). No patients had grade 4 or 5 AEs. In the present cohort, 132 (90.4%) patients underwent protocol bladder biopsy approximately 6 months after the initiation of intravesical BCG therapy. Prostatic urethra biopsy was carried out on 68 of 99 (68.7%) male patients. Table 3 summarizes biopsy outcomes according to the cystoscopic appearance and urine cytology. In the 17 patients with visible tumors cystoscopically, 11 had disease (four had pTa, six had pT1 and one had pT2), whereas six did not have disease recurrence. In the 73 patients with a normal appearance, three (4.1%, 3/73) had disease recurrence, including one (pT4, prostatic urethra) with negative cytology (1.4%, 1/70). Table S1 shows subsequent treatments in the 18 patients with disease at the time of protocol biopsy. Nine patients underwent immediate cystectomy.

**Table 3:** Results of protocol biopsy based on cystoscopic appearance and urine cytology

	Urine cytology						Total
	Positive		Suspicious		Negative/atypical		
	Biopsy positive	Biopsy negative	Biopsy positive	Biopsy negative	Biopsy positive	Biopsy negative	
Normal appearance, *n* (pT stage of recurrent disease)	1 (pT4: 1)	0	1 (pT1: 1)	1	1 (pT4: 1)	69	73
Abnormal appearance, *n* (pT stage of recurrent disease)	0	0	1 (pT1: 1)	0	3 (pTa: 1, pT1: 1, CIS: 1)	38	42
Visible tumor (+), *n* (pT stage of recurrent disease)	0	0	0	0	11 (pTa: 4, pT1: 6, pT2: 1)	6	17
Total	1	0	2	1	15	113	132

Total *n* = 132.

**Fig. 2:**
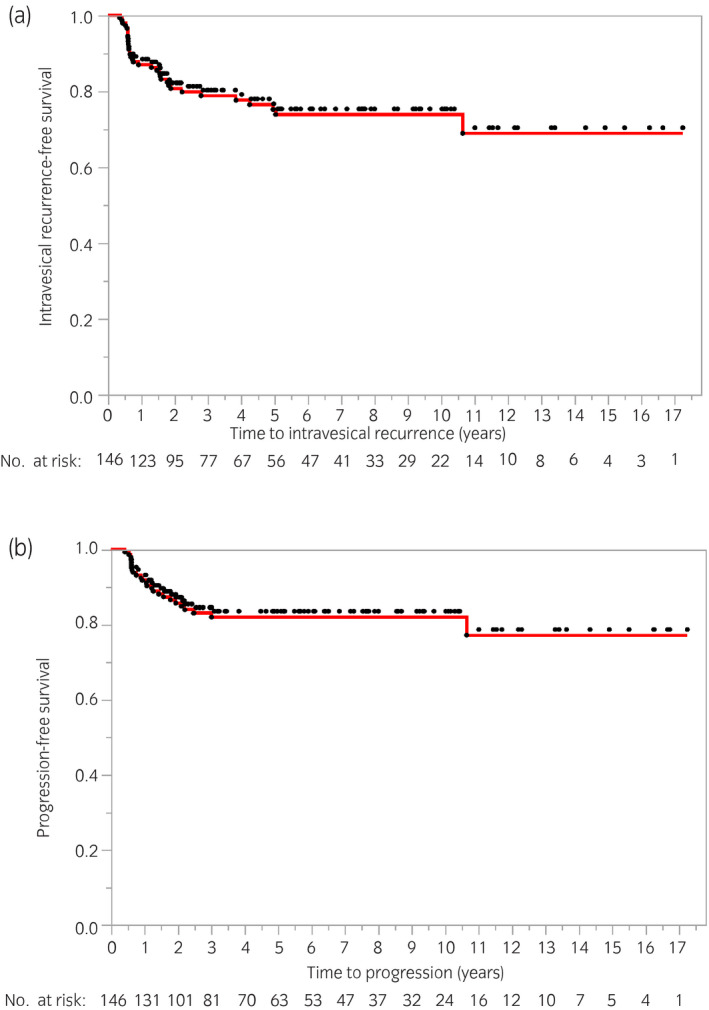
Kaplan–Meier survival analysis displaying times to (a) intravesical recurrence and (b) disease progression in all patients.

Figure 2 shows Kaplan–Meier curves of intravesical recurrence and disease progression in all patients. The median follow‐up time was 61 months (range 9–207 months). The 2‐ and 5‐year intravesical RFS rates were 80.7% and 75.2%, respectively (Fig. 2a). The 2‐ and 5‐year PFS rates were 85.7% and 82.0%, respectively (Fig. 2b).

**Fig. 3:**
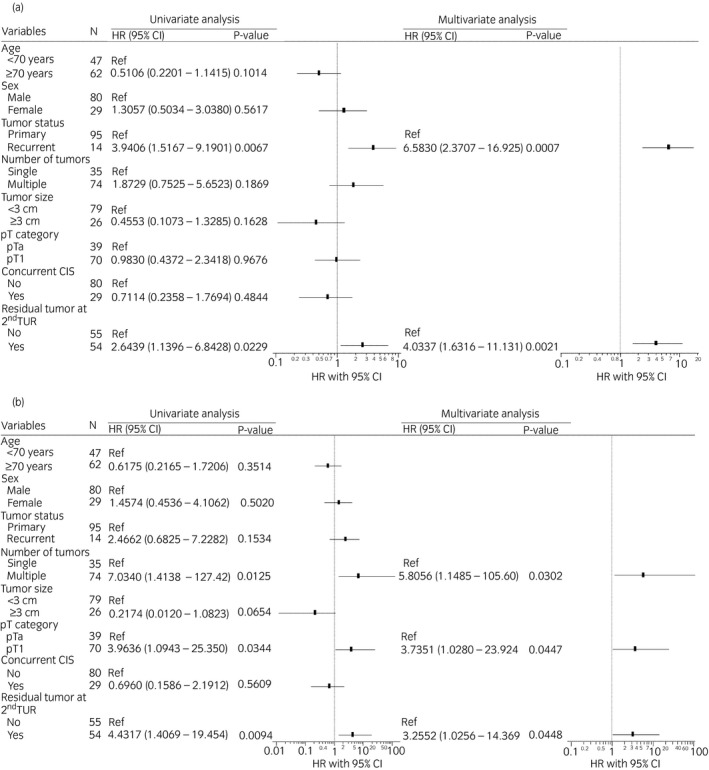
Univariate and multivariate Cox’s proportional hazard regression analyses predicting (a) intravesical recurrence and (b) disease progression.

**Fig. 4:**
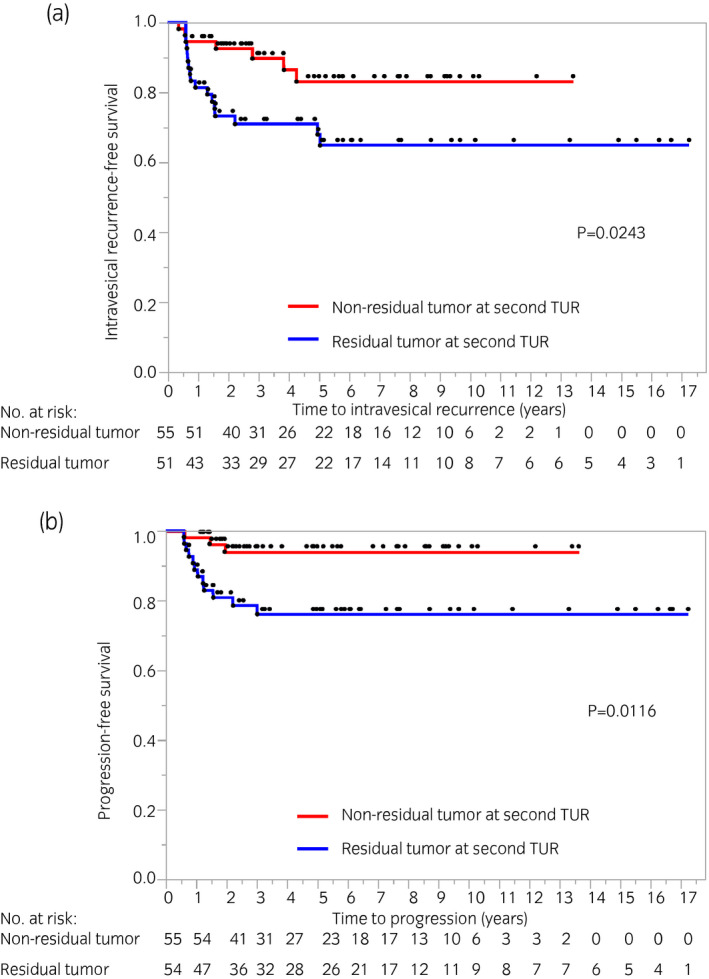
Kaplan–Meier survival analysis showing times to (a) disease recurrence and (b) invasive bladder cancer recurrence based on residual tumors at second TUR.

We investigated risk factors for intravesical recurrence after BCG induction therapy among patients who underwent second TUR (*n* = 109). Figure 3a shows univariate and multivariate Cox proportional hazards regression models on intravesical recurrence. Univariate and multivariate analyses showed that recurrent tumors (HR 6.583, *P* = 0.0007) and residual tumors at second TUR (HR 4.0337, *P* = 0.0021) were independent risk factors for intravesical recurrence. We also examined risk factors for disease progression. The multivariate analysis showed that multiple tumors, pT1 disease and residual tumors at the second TUR were associated with disease progression (*P* = 0.0302, *P* = 0.0447 and *P* = 0.0448 respectively, Fig. 3b). The 2‐ and 5‐year intravesical RFS rates were 92.5% and 83.1%, respectively, in patients without residual tumors at the second TUR, and were 73.2% and 68.0%, respectively, in those with residual tumors (*P* = 0.0243; Fig. 4a). In the group excluding concurrent CIS, the patients with residual tumors at second TUR had worse intravesical RFS rates (*P* = 0.0136; Fig. S1). The 2‐ and 5‐year PFS rates were both 94.0% in patients without residual tumors at second TUR, and were 80.8% and 76.1%, respectively, in those with residual tumors (*P* = 0.0116, Fig. 4b).

## Discussion

Several major guidelines recommend BCG induction therapy for high‐risk NMIBC patients,^2^, ^11^ and based on the prospective studies,^5^, ^12^, ^13^ maintenance BCG therapy was widely disseminated in real‐world clinical practice. Early discontinuation as a result of BCG toxicity and the current BCG shortage are major issues associated with maintenance therapy. Furthermore, the optimal instillation schedule remains controversial and the majority of previous studies did not include a second TUR in their protocols.^5^, ^12^, ^13^ In our hospital, we have been utilizing eight weekly instillations of BCG induction therapy without a maintenance schedule. Due to the importance of accurate disease staging and maximum disease elimination before BCG, we actively carried out a second TUR on high‐risk NMIBC patients. As shown in Table 2, residual disease was detected in 16 (41.0%) patients with pTa and in 39 (54.3%) with pT1 disease, which is consistent with previous findings.^14^, ^15^ Univariate and multivariate analyses showed that concurrent CIS was an independent risk factor for residual tumors at the second TUR (*P* = 0.0070, Fig. 1). Takaoka *et al*. also reported that among 73 high‐grade T1 bladder cancer patients, 37 (51%) had residual disease based on pathological findings from the second TUR, and identified the presence of concurrent CIS at the initial TUR as a risk factor for residual disease.^16^

A second TUR has been shown to enhance the effects of BCG therapy. Sfakianos *et al*. reported that 3 months after the completion of induction BCG (6 weekly instillations), the recurrence rate was 44.3% among patients who underwent single resection (*n* = 127) and 9.6% in those with a second TUR (*n* = 894; *P* < 0.01).^17^ Herr *et al*. also detected recurrent tumors in the first cystoscopy in 75 out of 132 patients (57%) undergoing a single TUR before the instillation of BCG, and in 62 (29%) after a second TUR (*n* = 215; *P* = 0.001).^18^ In the present study, 2‐ and 5‐year intravesical RFS rates were 80.7% and 75.2%, respectively, whereas 2‐ and 5‐year PFS rates were 85.7% and 82.0%, respectively. Approximately 80% of patients completed eight instillations. Intravesical RFS rates were higher in the present study that in previous maintenance BCG studies without a routine second TUR.^5^, ^6^, ^19^ Although the definition of progression varied among previous studies, ours was not an inferior rate. The present results underscore the importance of meticulous TUR before BCG instillation. The findings of the NIMBUS trial were recently reported;^20^ NIMBUS was the first prospective trial to include a routine second TUR in the protocol, and 91% of patients underwent a second TUR before BCG induction therapy. The NIMBUS study assessed whether a smaller number of BCG instillations (induction at 1, 2 and 6 weeks followed by 2 weeks of maintenance at 3, 6 and 12 months) achieved equivalent outcomes to the standard number of instillations (6 weekly instillations followed by 3 weeks of maintenance at 3, 6 and 12 months) in patients with high‐grade NMIBC. Disease recurrence was observed in 21 of 175 patients in the standard schedule cohort, and 46 of 170 in the reduced schedule cohort, and safety analyses showed that the reduced schedule was inferior to the standard schedule for the primary end‐point according to the previously used stop criterion. Although the median follow‐up time was short (12 months for all patients), 2‐year RFS rates were 85% in the standard schedule cohort and 66% in the reduced schedule cohort. Taken together with the present results, the NIMBUS trial showed the importance of TUR quality before BCG and adequate BCG induction. Although the present study did not confirm that the instillation of BCG eight times after routine second TUR was sufficient without a maintenance schedule, there is now a global BCG shortage. The American Urological Association has recommended several management approaches in the face of the current shortage (https://www.auanet.org/about‐us/bcg‐shortage‐info). For example, term No. 6 is “In the event of BCG supply shortage, maintenance therapy should not be given and BCG‐naïve patients with high‐risk disease should be prioritized for induction BCG.” We propose our BCG protocol (without a maintenance schedule) after thorough surgical resection as a treatment option.

In the present study, residual tumors at the second TUR were associated with intravesical recurrence and disease progression, which is consistent with previous findings.^16^, ^21^, ^22^, ^23^ Guevara *et al*. reported recurrence and progression rates of 11.4 (8/70) and 5.7% (4/70), respectively, in patients without residual tumors at the second TUR, and 27.7 (13/47) and 17.0% (8/47), respectively, in those with residual tumors among the maintenance BCG cohort.^23^ Takaoka *et al*. also found that 3‐year RFS rates were 91% in pT0 patients and 65% in those with pTis/a at the second TUR (*P* = 0.03).^16^ Taken together with the present results, a second TUR provides prognostic information on BCG outcomes, and careful follow ups are mandatory for patients with remaining disease at second TUR. In some cases, initial TUR is not of adequate quality, with residual tumor at second TUR. Mariappan *et al*. reported that good‐quality TUR (complete resection with detrusor muscle presence carried out by experienced surgeons) reduced early bladder recurrence.^24^ The present study also considered that the quality of TUR affects treatment outcomes in the BCG treatment group. Theoretically, a tumor‐free status is achieved after second TUR in most cases. Nevertheless, BCG outcomes were different with or without residual tumors at the second TUR. Tumors that are difficult to completely resect at initial TUR might be highly aggressive or prone to recurrence based on differences in molecular expression profiles. Therefore, further studies are required to elucidate the underlying mechanisms.

The BCG strain is another factor that might affect treatment outcomes. In the present cohort, approximately 80% of patients received BCG Tokyo‐172, whereas the others received BCG Connaught. Each BCG strain has extensive genotypic diversity, resulting in different immune responses.^25^, ^26^ Rentsch *et al*. reported that the BCG Connaught achieved significantly better RFS than BCG TICE in high‐risk NMIBC patients.^27^ A meta‐analysis showed that disease recurrence rates were slightly lower with the BCG Tokyo‐172 than with BCG TICE, RIVM, Pasteur and Connaught.^28^

In our hospital, post‐BCG bladder biopsy is regularly carried out approximately 6 months after BCG induction therapy. As shown in Table 3, three (4.1%) out of the 73 patients with negative cystoscopy had disease recurrence, whereas just one (1.4%) out of the 70 patients with negative cystoscopy and negative/atypical cytology had disease, which is consistent with previous findings.^29^ Therefore, routine bladder biopsy needs to be tailored according to cystoscopy and cytology findings.

There were several limitations to the present study. This was a retrospective analysis with a relatively small cohort. Many surgeons were involved in TUR, with some cases resulting in insufficient quality; however, the attending surgeons always supervised surgeons where necessary. A central pathology review was not carried out. Nevertheless, the present results underscore the importance of TUR quality before BCG therapy, and several important results were obtained.

In conclusion, routine second TUR followed by eight weekly instillations of BCG induction therapy showed good disease control. However, physicians should have sufficient discussions with patients on the merit and demerit of BCG maintenance strategy, and provide them with relevant information. Currently in Japan, due to the Tokyo‐172 strain, we are not facing BCG shortage. However, BCG shortage is still a significant concern in Western countries, and we believe that accurate disease staging and elimination by second TUR could maximize BCG treatment benefits, and become an alternative to maintenance instillations. The present results suggest the importance of complete disease elimination before BCG instillation, and our no‐maintenance strategy in conjunction with second TUR has potential as a treatment option in the face of the current BCG shortage. Patients with risk factors for intravesical recurrence and disease progression, such as residual tumors at second TUR, need to be carefully followed up after induction courses of BCG.

## Author contributions

Hiroshi Kikuchi: Data curation; Formal analysis; Investigation; Methodology; Writing – original draft; Writing – review & editing. Takashige Abe: Conceptualization; Project administration; Validation; Writing – original draft; Writing – review & editing. Ryuji Matsumoto: Investigation. Takahiro Osawa: Formal analysis; Investigation. Satoru Maruyama: Data curation; Investigation. Sachiyo Murai: Investigation. Nobuo Shinohara: Project administration; Supervision.

## Conflict of interest

None declared.

## Approval of the research protocol by an Institutional Reviewer Board

The protocol for this research project has been approved by a suitably constituted ethics committee of the institution and it conforms to the provisions of the Declaration of Helsinki.

## Informed consent

All participants provided written informed consent with guarantees of confidentiality.

## Registry and the Registration No. of the study/trial

Institutional Review Board of Hokkaido University Hospital, Approval No. 019‐0092.

## Animal studies

N/A.

## Supporting information


**Figure S1.** Kaplan–Meier survival analysis showing times to disease recurrence in the group excluding concurrent CIS.
**Table S1.** Treatment course of biopsy‐positive patients.

## References

[1] Kamat AM , Hahn NM , Efstathiou JA *et al*. Bladder cancer. Lancet 2016; 388: 2796–810. 27345655 10.1016/S0140-6736(16)30512-8

[2] Babjuk M , Burger M , Compérat EM *et al*. European Association of Urology Guidelines on non‐muscle‐invasive Bladder Cancer (TaT1 and carcinoma in situ) – 2019 update. Eur. Urol. 2019; 76: 639–57. 31443960 10.1016/j.eururo.2019.08.016

[3] Shelley MD , Kynaston H , Court J *et al*. A systematic review of intravesical bacillus Calmette–Guerin plus transurethral resection vs transurethral resection alone in Ta and T1 bladder cancer. BJU Int. 2001; 88: 209–16. 11488731 10.1046/j.1464-410x.2001.02306.x

[4] Malmstrom PU , Sylvester RJ , Crawford DE *et al*. An individual patient data meta‐analysis of the long‐term outcome of randomised studies comparing intravesical mitomycin C versus bacillus Calmette–Guerin for non‐muscle‐invasive bladder cancer. Eur. Urol. 2009; 56: 247–56. 19409692 10.1016/j.eururo.2009.04.038

[5] Lamm DL , Blumenstein BA , Crissman JD *et al*. Maintenance bacillus Calmette–Guerin immunotherapy for recurrent TA, T1 and carcinoma in situ transitional cell carcinoma of the bladder: a randomized Southwest Oncology Group Study. J. Urol. 2000; 163: 1124–9. 10737480

[6] Oddens J , Brausi M , Sylvester R *et al*. Final results of an EORTC‐GU cancers group randomized study of maintenance bacillus Calmette–Guérin in intermediate‐ and high‐risk Ta, T1 papillary carcinoma of the urinary bladder: one‐third dose versus full dose and 1 year versus 3 years of maintenance. Eur. Urol. 2013; 63: 462–72. 23141049 10.1016/j.eururo.2012.10.039

[7] Martínez‐Piñeiro L , Portillo JA , Fernández JM *et al*. Maintenance therapy with 3‐monthly bacillus Calmette–Guérin for 3 years is not superior to standard induction therapy in high‐risk non–muscle‐invasive urothelial bladder carcinoma: final results of randomised CUETO study 98013. Eur. Urol. 2015; 68: 256–62. 25794457 10.1016/j.eururo.2015.02.040

[8] Chen S , Zhang N , Shao J , Wang X . Maintenance versus non‐maintenance intravesical bacillus Calmette–Guerin instillation for non‐muscle invasive bladder cancer: a systematic review and meta‐analysis of randomized clinical trials. Int. J. Surg. 2018; 52: 248–57. 29499363 10.1016/j.ijsu.2018.02.045

[9] Quan Y , Jeong CW , Kwak C , Kim HH , Kim HS , Ku JH . Dose, duration and strain of bacillus Calmette–Guerin in the treatment of nonmuscle invasive bladder cancer: meta‐analysis of randomized clinical trials. Medicine 2017; 96: e8300. 29049231 10.1097/MD.0000000000008300 PMC5662397

[10] Common Terminology Criteria for Adverse Events version 5.0. [Cited 31 Jan 2021.] Available from URL: https://ctep.cancer.gov/protocolDevelopment/electronic_applications/ctc.htm#ctc_50

[11] Chang SS , Boorjian SA , Chou R *et al*. Diagnosis and treatment of non‐muscle invasive bladder cancer: AUA/SUO guideline. J. Urol. 2016; 196: 1021–9. 27317986 10.1016/j.juro.2016.06.049

[12] Martinez‐Pineiro L , Portillo JA , Fernandez JM *et al*. Maintenance therapy with 3‐monthly bacillus Calmette–Guerin for 3 years is not superior to standard induction therapy in high‐risk non‐muscle‐invasive urothelial bladder carcinoma: final results of randomised CUETO study 98013. Eur. Urol. 2015; 68: 256–62. 25794457 10.1016/j.eururo.2015.02.040

[13] Oddens J , Brausi M , Sylvester R *et al*. Final results of an EORTC‐GU cancers group randomized study of maintenance bacillus Calmette–Guerin in intermediate‐ and high‐risk Ta, T1 papillary carcinoma of the urinary bladder: One‐third dose versus full dose and 1 year versus 3 years of maintenance. Eur. Urol. 2013; 63: 462–72. 23141049 10.1016/j.eururo.2012.10.039

[14] Cumberbatch MGK , Foerster B , Catto JWF *et al*. Repeat transurethral resection in non‐muscle‐invasive bladder cancer: a systematic review. Eur. Urol. 2018; 73: 925–33. 29523366 10.1016/j.eururo.2018.02.014

[15] Gordon PC , Thomas F , Noon AP , Rosario DJ , Catto JWF . Long‐term outcomes from re‐resection for high‐risk non‐muscle‐invasive bladder cancer: a potential to rationalize use. Eur. Urol. Focus 2019; 5: 650–7. 29089252 10.1016/j.euf.2017.10.004

[16] Takaoka E , Matsui Y , Inoue T *et al*. Risk factors for intravesical recurrence in patients with high‐grade T1 bladder cancer in the second TUR era. Jpn. J. Clin. Oncol. 2013; 43: 404–9. 23444116 10.1093/jjco/hyt016

[17] Sfakianos JP , Kim PH , Hakimi AA , Herr HW . The effect of restaging transurethral resection on recurrence and progression rates in patients with nonmuscle invasive bladder cancer treated with intravesical bacillus Calmette–Guerin. J. Urol. 2014; 191: 341–5. 23973518 10.1016/j.juro.2013.08.022 PMC4157345

[18] Herr HW . Restaging transurethral resection of high risk superficial bladder cancer improves the initial response to bacillus Calmette–Guerin therapy. J. Urol. 2005; 174: 2134–7. 16280743 10.1097/01.ju.0000181799.81119.fc

[19] Sylvester RJ , Brausi MA , Kirkels WJ *et al*. Long‐term efficacy results of EORTC genito‐urinary group randomized phase 3 study 30911 comparing intravesical instillations of epirubicin, bacillus Calmette–Guérin, and bacillus Calmette–Guérin plus isoniazid in patients with intermediate‐ and high‐risk. Eur. Urol. 2010; 57: 766–73. 20034729 10.1016/j.eururo.2009.12.024 PMC2889174

[20] Grimm M‐O , Van Der Heijden AG , Colombel M *et al*. Treatment of high‐grade non–muscle‐invasive bladder carcinoma by standard number and dose of BCG instillations versus reduced number and standard dose of BCG instillations: results of the European Association of Urology Research Foundation randomised phase III clinical trial “NIMBUS”. Eur. Urol. 2020; 78: 690–8. 32446864 10.1016/j.eururo.2020.04.066

[21] Sanseverino R , Napodano G , Campitelli A , Addesso M . Prognostic impact of ReTURB in high grade T1 primary bladder cancer. Arch. Ital. Urol. Androl. 2016; 88: 81–5. 27377079 10.4081/aiua.2016.2.81

[22] Tae BS , Jeong CW , Kwak C , Kim HH , Moon KC , Ku JH . Pathology in repeated transurethral resection of a bladder tumor as a risk factor for prognosis of high‐risk non‐muscle‐invasive bladder cancer. PLoS One 2017; 12: e0189354. 29244843 10.1371/journal.pone.0189354 PMC5731735

[23] Guevara A , Salomon L , Allory Y *et al*. The role of tumor‐free status in repeat resection before intravesical bacillus Calmette–Guerin for high grade Ta, T1 and CIS bladder cancer. J. Urol. 2010; 183: 2161–4. 20399454 10.1016/j.juro.2010.02.026

[24] Mariappan P , Finney SM , Head E *et al*. Good quality white‐light transurethral resection of bladder tumours (GQ‐WLTURBT) with experienced surgeons performing complete resections and obtaining detrusor muscle reduces early recurrence in new non‐muscle‐invasive bladder cancer: validation across time and place and recommendation for benchmarking. BJU Int. 2012; 109: 1666–73. 22044434 10.1111/j.1464-410X.2011.10571.x

[25] Gan C , Mostafid H , Khan MS , Lewis DJ . BCG immunotherapy for bladder cancer–the effects of substrain differences. Nat. Rev. Urol. 2013; 10: 580–8. 24042563 10.1038/nrurol.2013.194

[26] Ritz N , Hanekom WA , Robins‐Browne R , Britton WJ , Curtis N . Influence of BCG vaccine strain on the immune response and protection against tuberculosis. FEMS Microbiol. Rev. 2008; 32: 821–41. 18616602 10.1111/j.1574-6976.2008.00118.x

[27] Rentsch CA , Birkhäuser FD , Biot C *et al*. Bacillus Calmette–Guérin strain differences have an impact on clinical outcome in bladder cancer immunotherapy. Eur. Urol. 2014; 66: 677–88. 24674149 10.1016/j.eururo.2014.02.061

[28] Boehm BE , Cornell JE , Wang H , Mukherjee N , Oppenheimer JS , Svatek RS . Efficacy of bacillus Calmette–Guérin strains for treatment of nonmuscle invasive bladder cancer: a systematic review and network meta‐analysis. J. Urol. 2017; 198: 503–10. 28286068 10.1016/j.juro.2017.01.086 PMC6464123

[29] Swietek N , Waldert M , Rom M *et al*. The value of transurethral bladder biopsy after intravesical bacillus Calmette–Guerin instillation therapy for nonmuscle invasive bladder cancer: a retrospective, single center study and cumulative analysis of the literature. J. Urol. 2012; 188: 748–53. 22819422 10.1016/j.juro.2012.05.015

