# Supplementary material for: Outcomes of bacillus Calmette–Guérin therapy without a maintenance schedule for high‐risk non‐muscle‐invasive bladder cancer in the second transurethral resection era
Source: Int J Urol. 2021 Dec 11;29(3):251–8. doi: 10.1111/iju.14761 (PMC9299795; doi:10.1111/iju.14761)
Supplement: Supplementary file 1 — Figure S1. Kaplan–Meier survival analysis showing times to disease recurrence in the group excluding concurrent CIS. Table S1. Treatment course of biopsy‐positive patients. [file IJU-29-251-s001.pdf]

Supplementary Table. Treatment course of biopsy-positive patients

Tumor residual case in protocol biopsy without visible tumor (N=7)

Case	Age	Sex	Cytology	Pathology at 1st TUR	Pathology at protocol biopsy	Additional treatment	Outcome
1	71	M	Atypical	G3T1withCIS	CIS	2nd BCG→Chemotherapy for metastatic disease	Arrive with tumor
2	70	M	Negative	G3T1withCIS	G2T4 (prostate invasion)	Cystectomy	Arrive without tumor
3	55	M	Positive	G3Ta	G3T4 (prostate invasion)	Cystectomy	Arrive without tumor
4	69	M	Negative	G3Ta with CIS	G3T1	Cystectomy	Arrive without tumor
5	43	F	Suspicious	G3Ta with CIS	G3T1	Cystectomy	Arrive without tumor
6	59	M	Suspicious	CIS	G3T1	Cystectomy	Arrive without tumor
7	68	M	Negative	G3T1	G3Ta	Cystectomy	Cancer death

Tumor residual case in protocol biopsy with visible tumor (N=11)

Case	Age	Sex	Cytology	Pathology at 1st TUR	Pathology at protocol biopsy	Additional treatment	Outcome
1	67	M	Negative	G2Ta	G2Ta	Observe	Arrive without tumor
2	64	M	Atypical	G3T1	G3T1	Cystectomy	Arrive without tumor
3	80	M	Negative	G3T1	G3T1	Observe	Metastasis (no treatment)
4	69	M	Atypical	CIS	G3T1	2nd BCG	Arrive without tumor
5	75	M	Negative	G2Ta	G2Ta	2nd BCG	Arrive without tumor
6	83	F	Negative	G2T1	G3T1	Observe	Arrive without tumor
7	82	M	Negative	G3Ta with CIS	G3T2+ low grade B-cell lymphoma	Chemoradiation	Death from other causes
8	64	F	Atypical	G3T1	G3T1	Cystectomy	Arrive without tumor
9	64	F	Negative	G3T1	G3T1	Cystectomy	Unknown
10	84	F	Negative	G2Ta	G2Ta	MMC→G2Ta recurrence →TUR	Arrive without tumor
11	42	M	Negative	G3T1	High Ta	Observe→G3T1 recurrence →cystectomy	Arrive without tumor

Supplementary Figure. Kaplan-Meier survival analysis displaying times to disease recurrence in the group excluding concurrent CIS.

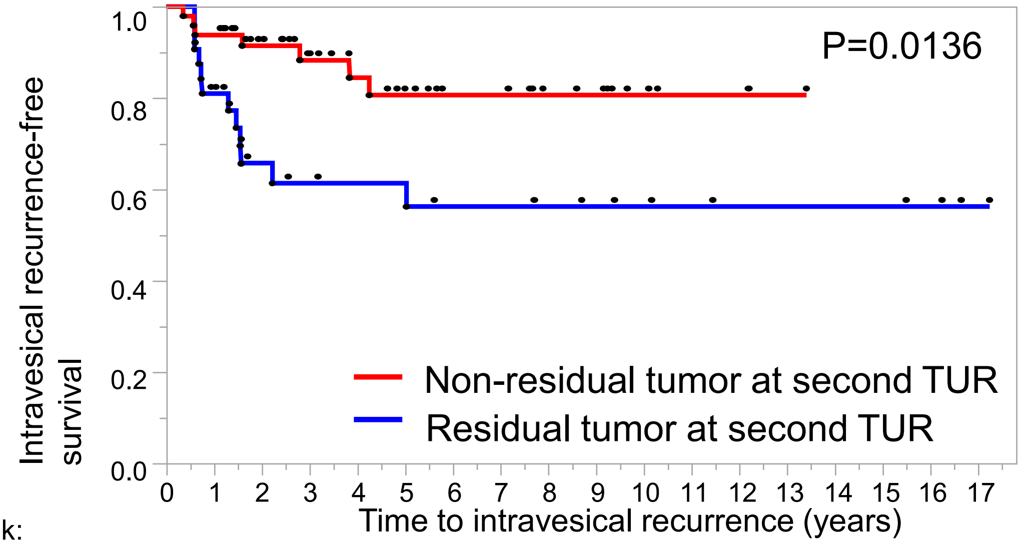

No. at risk:																		
Non-residual tumor	48	45	34	27	22	18	14	14	10	9	5	2	2	1	0	0	0	0
Residual tumor	32	24	15	13	12	12	10	10	8	7	6	5	4	4	4	4	3	1
